# A multidimensional strategy for uncovering comprehensive quality markers of *Scutellariae Radix* based on UPLC-Q-TOF-MS analysis, artificial neural network, network pharmacology analysis, and molecular simulation

**DOI:** 10.3389/fpls.2024.1423678

**Published:** 2024-07-03

**Authors:** Meiqi Liu, Xiaoran Zhao, Jinli Wen, Lili Sun, Rui Huang, Huijie Zhang, Yi Liu, Xiaoliang Ren

**Affiliations:** ^1^ School of Chinese Materia Medica, Tianjin University of Traditional Chinese Medicine, Tianjin, China; ^2^ Department of Pharmacy, Tianjin Academy of Traditional Chinese Medicine Affiliated Hospital, Tianjin, China; ^3^ Chinese Medicine Research Institute, Tianjin Academy of Traditional Chinese Medicine, Tianjin, China

**Keywords:** *Scutellariae Radix*, quality marker, artificial neural network, UPLC-Q-TOF-MS, molecular simulation

## Abstract

**Introduction:**

*Scutellariae Radix* (SR), derived from the root of *Scutellaria baicalensis* Georgi, is a traditional Chinese medicine (TCM) for clearing heat and cooling blood. It has been used as a traditional herbal medicine and is popular as a functional food in Asian countries today.

**Methods:**

In this study, UPLC-Q-TOF-MS was first employed to identify the chemical components in the ethanol extract of SR. Then, the extraction process was optimized using star point design-response surface methodology. Fingerprints of different batches and processed products were established, and chemical markers were screened through a combination of various artificial neural network models. Finally, network pharmacology and molecular simulation techniques were utilized for verification to determine the quality markers.

**Results:**

A total of 35 chemical components in SR were identified, and the optimal extraction process was determined as follows: ultrasonic extraction with 80% methanol at a ratio of 120:1 for 70 minutes, with a soaking time of 30 minutes. Through discriminant analysis using various artificial neural network models, the samples of SR could be classified into two categories based on their growth years: Kuqin (dried roots of older plants) and Ziqin (roots of younger plants). Moreover, the samples within each category could be further clustered according to their origins. The four different processed products of SR could also be distinguished separately. Finally, through the integration of network pharmacology and molecular simulation techniques, it was determined that baicalin, baicalein, wogonin, norwogonin, norwogonin-8-O-glucuronide, skullcapflavone II, hispidulin, 8, 8"-bibaicalein, and oroxylin A-7-O-beta-D-glucuronide could serve as quality markers for SR.

**Discussion:**

The primary factors affecting the quality of SR were its growth years. The geographic origin of SR was identified as a secondary factor affecting its quality. Processing also had a significant impact on its quality. The selected quality markers have laid the foundation for the quality control of SR, and this research strategy also provides a research paradigm for improving the quality of TCM.

## 1 Introduction

*Scutellariae Radix* (SR), derived from Scutellaria baicalensis Georgi’s root, is widely used in China for treating fever, ulcers, bronchitis, hepatitis, and inflammatory symptoms (Huang et al., 2023). SR contains various components such as flavonoids, phenolic glycosides, alcohol glycosides, volatile oils, trace elements, and amino acids (Zhao et al., 2019b). It exhibit antibacterial, anti-tumor, anti-inflammatory, anti-viral, anti-oxidation, anti-allergy, anti-fatigue, cardiovascular and cerebrovascular protection, and other pharmacological activities (Liu et al., 2013; Wang et al., 2018; Song et al., 2020; Liao et al., 2021; Liu et al., 2022a). According to the different growth years of SR, Tao Hongjing divided the herbs into two kinds, and thought that “dark and solid ones are good”. In SR, those harvested in two years generally have firmer roots and are called Ziqin (ZQ), while those harvested in more than three years tend to have hollow old roots and are referred to as Kuqin (KQ). Consequently, the differentiation between KQ and ZQ essentially represents the distinction in the varying growth years of SR (Yang et al., 2017; Sun et al., 2023). However, due to the limitations of research, most of the current medical units do not strictly differentiate between the use of SR and its commercial specifications, which hinders the inheritance and application of the connotations of traditional Chinese medicine (TCM). Moreover, due to China’s vast area, the SR planted in various production areas exhibits significant differences in chemical composition, influenced by factors such as geographic environment, temperature, and climate (Cao et al., 2019). Consequently, when assessing the quality of SR, the place of origin is a crucial aspect that cannot be overlooked.

The processing methods of SR have been documented in literature from past dynasties. In ancient times, the primary processing method was stir-frying, often accompanied by various auxiliary materials like wine, vinegar, ginger, rice swill, and pig bile. Raw SR is bitter and cold, possessing a strong ability to dissipate heat and detoxify toxins. When roasted in wine, it tempers its bitter and cold properties, directing the medicine upwards, often used to clear lung heat and dampness and heat on the muscle surface of the limbs. Additionally, frying into charcoal products helps alleviate heat and diarrhea, making it a common choice for clearing such symptoms (Huang et al., 2020a; Hu et al., 2020). Modern processing methods of SR include net processing, cutting, stir-frying, wine stir-frying, honey stir-frying, carbon stir-frying, vinegar stir-frying, and others (Wang et al., 2017; Huang et al., 2020a; Huang et al, 2020b). Current research on SR mainly focuses on the pharmacological and clinical effects among the concoctions, and there is a paucity of research on the differences in composition and quality evaluation.

Despite the numerous modern studies conducted on the quality of SR, the traditional methods for evaluating its quality are inadequate, failing to provide a comprehensive assessment. For example, traditional methods such as microscopic identification, thin-layer chromatography, and the detection of the content of a single or several components are insufficient to accurately reflect the herb’s overall quality, and the operation procedures are relatively cumbersome. Multiple factors, such as geographical region, species variation, harvesting time, and processing methods, can significantly influence the composition of these components in natural herbs (Sun et al., 2018). Consequently, ensuring quality assurance in research becomes a challenging task. It is essential to establish sensitive and accurate methods to control the quality of natural samples. Developing the concept of a “quality marker” (Q-Marker) lays the foundation for establishing an independent process quality control system for herbal products (Liu et al., 2018; Zhang et al., 2022). In addition, a wide range of analytical instruments and techniques, including ultra-performance liquid chromatography coupled with quadrupole time-of-flight mass spectrometry (UPLC-Q-TOF-MS), fingerprinting, network pharmacology, molecular docking, chemometrics, and artificial neural networks (ANNs), can furnish the means and ideas for discovering quality control indices and enhancing the quality evaluation system of TCM.

Fingerprint plays an important role in the process of multi-component analysis of TCM and is widely used in the quality control and quality evaluation. UPLC fingerprint, with its high degree of separation and short analytical time, is widely used in many fields such as TCM, food, and chemical industry (Liang et al., 2010). However, the chemical composition of TCM is complex, and there are many problems in fingerprinting research, such as baseline drift, peak overlap and other common problems of chromatographic analysis, which limit the application of fingerprinting in quality control (Zhou et al., 2020; Liu et al., 2022b). ANN can be used to solve the common problems in the fingerprinting of TCM through statistical or mathematical methods to establish a link between the measured value of the chemical system and the state of the system, and can provide a variety of analytical methods for identification. The combination of ANN and fingerprinting has important scientific value and practical significance in the quality control and evaluation of TCM (Yang et al., 2024). And the complex and diverse chemical compositions of TCM are characterized by multi-components, multi-targets, and multi-pathways in the process of ameliorating the diseases of the organism, which is compatible with the ideas and concepts of network pharmacology (Wang et al., 2021; Zhao et al., 2023). Molecular docking technology can simulate receptor-ligand interactions based on the computer level, providing a means to predict the binding ability of compounds to key target proteins *in vivo* (Pinzi and Rastelli, 2019). Therefore, network pharmacology combined with molecular docking can provide ideas and means to reveal the mechanism of action of TCM and explore the material basis of their efficacy.

This study employed a combination of UPLC-Q-TOF-MS combined fingerprint, ANN, screening of characteristic components, network pharmacology, and molecular simulation to evaluate the quality of SR comprehensively. Several factors affecting the quality of SR were evaluated, including growth years, origin and processing methods. And the quality markers are screened out according to these factors. This research strategy has established a solid foundation for the quality assessment of TCM and has offered a research framework for the standardization of TCM.

## 2 Material and methods

### 2.1 Materials and reagents

**Figure 1:**
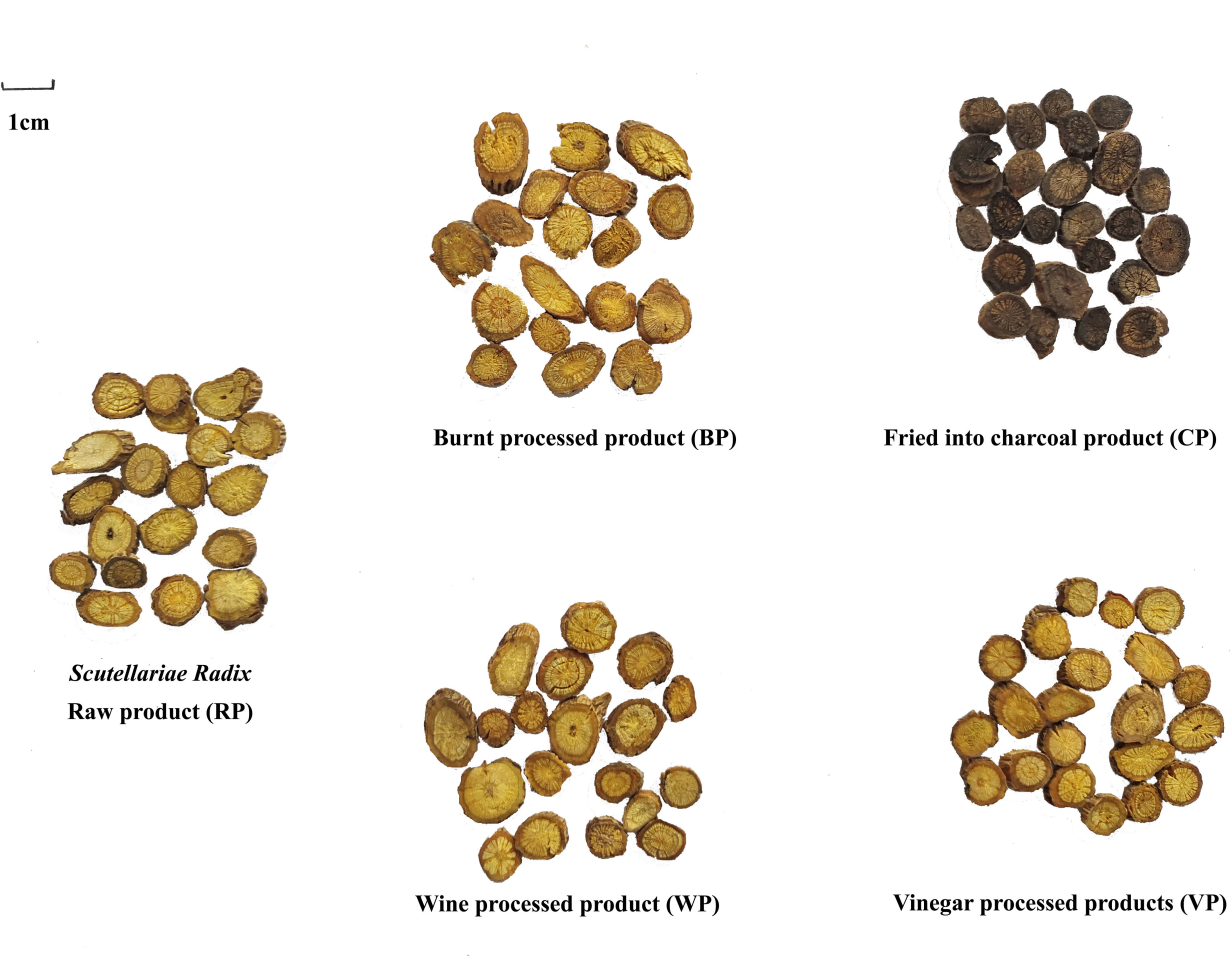
Different processed products of SR.

48 batches of SR were collected from different areas in China (**Supplementary Table S1**). Dr. Lin Ma identified the voucher specimens according to the Pharmacopeia of the People’s Republic of China (2020 edition). Different processed products were processed and manufactured according to the standards of the Science of Processing of TCM, the National Standard for the Processing of TCM, and the Pharmacopoeia of the People’s Republic of China. 5 copies of each type of processed product were processed in parallel, with a total of 25 batches of different processed product samples including raw products. Specific sample information was listed in **Tables 1** and **2**. Four kinds of processed products (fried into charcoal product (CP), burnt processed product (BP), wine processed product (WP), vinegar processed product (VP)) and raw products were shown in **Figure 1**.

**Table 1:** Information of 48 batches of SR.

Batches	Origin	Batches	Origin	Batches	Origin	Batches	Origin
Q1	Shanxi	Q13	Neimenggu	Q25	Shanxi	Q37	Neimenggu
Q2	Shanxi	Q14	Hebei	Q26	Neimenggu	Q38	Neimenggu
Q3	Shanxi	Q15	Neimenggu	Q27	Neimenggu	Q39	Neimenggu
Q4	Shanxi	Q16	Neimenggu	Q28	Neimenggu	Q40	Neimenggu
Q5	Neimenggu	Q17	Shanxi	Q29	Shanxi	Q41	Neimenggu
Q6	Shanxi	Q18	Shanxi	Q30	Shanxi	Q42	Neimenggu
Q7	Shanxi	Q19	Hebei	Q31	Neimenggu	Q43	Neimenggu
Q8	Shanxi	Q20	Hebei	Q32	Shanxi	Q44	Shanxi
Q9	Shanxi	Q21	Shanxi	Q33	Shanxi	Q45	Shanxi
Q10	Shanxi	Q22	Shanxi	Q34	Shanxi	Q46	Shanxi
Q11	Shanxi	Q23	Shanxi	Q35	Neimenggu	Q47	Neimenggu
Q12	Shanxi	Q24	Shanxi	Q36	Neimenggu	Q48	Neimenggu

**Table 2:** Four processing techniques of SR.

Processing method	Specific processing technology
Fried into charcoal product (CP)	Take 100g of SR, place it in a preheated frying container, heat with high heat until the surface turns dark brown and the inside turns burnt yellow, remove it, spray a little clean water to extinguish any sparks.
Burnt processed product (BP)	Take 100g of SR, place it in a preheated frying container, heat it over gentle fire until the surface turns dark yellow and the color inside deepens.
Wine processed product (WP)	Take 100g of SR, add 10g of wine and mix well. Cover and let it sit for a moment until the wine is fully absorbed. Then, place it in a preheated frying container and heat it over a gentle fire until the surface of the medicine is slightly dry and dark yellow.
Vinegar processed product (VP)	Take 100g of SR, add 20 g of rice vinegar and mix well. Cover and let it marinate until the vinegar is fully absorbed. Then, place it into a preheated stir-frying container, heat over a gentle fire, and stir-fry until the surface of the medicine is slightly dry.

Baicalin (lot number: G07S11L123706, >98%) was purchased from Shanghai Yuanye Biotechnology Co., Ltd (Shanghai, China). Wogonoside (lot number: wkq22091502, >98%), wogonin (lot number: wkq 23011603, >98%), baicalein (lot number: wkq23021601, >98%), scutellarin (lot number: wkq 23041810, >98%), were purchased from Sichuan Vicchi Biotechnology Co., LTD. (Sichuan, China). LC-MS grade methanol, acetonitrile, and formic acid were purchased from Fisher Scientific (Pittsburgh, PA, USA). Distilled water was purchased from Watson Group Ltd (Hong Kong, China). All other reagents and solvents were of analytical grade.

### 2.2 Qualitative analysis of chemical constituents of SR based on UPLC - Q-TOF/MS

#### 2.2.1 Chromatographic conditions

UPLC-Q-TOF-MS analysis was conducted on a Waters Acquity UPLC system equipped with a Xevo G2-XS Q-TOF mass spectrometer (Waters, Milford, MA, USA). The chromatographic separation was performed on an ACQUITY UPLC BEH shield RP_18_ column (100 mm × 2.1 mm, 1.7 mm particle size; Waters), operated at 35°C. The mobile phase comprised water containing 0.2% formic acid (Solvent A) and acetonitrile (Solvent B). A gradient elution program was employed as follows: 0–6 minutes (18–20%, B), 6–8 minutes (20–22%, B), 8–20 minutes (22–25%, B), 20–28 minutes (25–50%, B), 28–30 minutes (50–60%, B), with a mobile phase flow rate of 0.2 mL/min. The detection wavelength was 254 nm, and the injection volume was 2 μL.

#### 2.2.2 Mass spectrometry conditions

The MS analysis was performed using an electrospray ionization source in positive/negative-ion mode, and the range of full-scan mass was 100–1000 Da. Source temperature and desolvation temperature were 120°C and 450°C, respectively. The desolvation gas flow was set at 800 L/h. The voltage of the capillary and cone were set at 3.0 kV and 40 V, respectively. All solutions were further filtered through a 0.22-μm nylon membrane prior to injection into the UPLC and UPLC-Q-TOF-MS systems.

#### 2.2.3 Preparation of sample

A sample powder (passed through a 24-mesh sieve) of 0.1 g was accurately weighed and placed in a stoppered conical flask. Precisely 8 mL of 80% methanol was added, and the mixture was sonicated for 30 min (power 400 W, frequency 40 kHz). After shaking, the mixture was centrifuged at 8000 rpm for 5 min, and the supernatant was collected. The solution was filtered through a 0.22 μm membrane before collecting the filtrate.

### 2.3 Optimization of ultrasonic extraction processing technology of SR

#### 2.3.1 Sample preparation

Separately, 5.0 mg of baicalein, baicalin, wogonoside, and wogonin were weighed as reference substances. In a brown volumetric flask, methanol was added to make up a total volume of 10 mL. The flask was shaken well to obtain the solution. The composite score was calculated as an indicator using the content proportion of the four indicators as weighting coefficients.

#### 2.3.2 Method validation

Six different concentrations were used to evaluate the linearity of the developed method under the optimal separation conditions. The calibration curves of baicalin, baicalein, wogonin, and wogonoside were constructed by plotting the peak areas (y) versus the concentrations (x). According to 100% of the mass percentage, the four substances were added into SR (0.1 g) respectively, the sample recovery and RSD % of the five compounds in samples were determined.

#### 2.3.3 Single factor experiment

The precise weighing of SR powder at 0.1g (Q1) was conducted, along with an investigation into the effects of methanol concentration, liquid-to-material ratio, soaking time, and ultrasonication time on the comprehensive score of four target components (baicalein, baicalin, wogonoside, and wogonin) (**Supplementary Table S1**). The weight coefficient was defined as the average value of a certain compound content divided by the sum of the average values of all compound contents. As a result, the comprehensive score was calculated as the sum of the products of each compound’s content and its corresponding weight coefficient.

#### 2.3.4 Response surface methodology for process optimization analysis

Based on the Box-Behnken central composite design principle, this experiment employs a response surface analysis method with four factors and three levels, building upon the foundation of single-factor experiments to explore the optimal methanol ultrasonic extraction process for SR. The factors and levels of the response surface analysis were shown in **Supplementary Table S2**. The response value was regressed and fitted with various aspects to obtain the regression equation. The interaction contour and 3D surface plot were drawn using Design-Expert software (V8.0.6.1).

### 2.4 Establish UPLC fingerprint for raw and processed products of SR

According to the chromatographic conditions under “2.2.1”, the reference substance and test solution were prepared for analysis.

#### 2.4.1 Method validation

Concerning intraday precision, intraday stability, and repeatability, the UPLC fingerprint method analysis was validated by referring to the national standard of TCM fingerprints (SFDA, 2000). The intraday precision variations were determined by continuously analyzing the six replicate sample solutions (Q1) on the same day. The intraday stability test was assessed by analyzing the same sample solution (Q1) at different time intervals (0, 1, 2, 4, 8, 12, and 24 h). Six sample repeats were prepared in parallel, and the repeatability of the UPLC method was calculated.

#### 2.4.2 Similarity evaluation

The data of 48 batches of raw SR and five kinds of 25 batches of different processed products of SR were imported into the “Assessment System for Similarity of Chromatographic Fingerprints of TCM (2012 Version A)”. Then Q1 was taken as the reference, the control map was established by the median method, the time window width was set to 0.1 min, and the fingerprint was based on multipoint correction mark peak matching. And the similarity evaluation result was obtained.

### 2.5 Multivariate chemometric analysis

The data management function of the liquid phase workstation was used to obtain the peak area, retention time, and other relevant information for 48 batches of raw products and four types of processed products. The obtained data matrix of the 48 batches of raw product samples (96 samples x 34 variables) and the sample matrix of the four types of processed products (25 samples x 34 variables) were separately imported into analysis software. Partial least squares discriminant Analysis (PLS-DA) and hierarchical cluster analysis (HCA) were performed using the Simca-p (Simca Imola SC, Imola, Bologna, Italy). Principal component analysis (PCA) and counter-propagation artificial neural network (CP-ANN) were performed using the Matlab R2018b (MathWorks Inc., Natick, MA, USA).

### 2.6 Target network analysis

The chemical markers that may affect the quality of SR predicted by neural networks were further screened in the TCMSP database (http://lsp.nwu.edu.cn/tcmsp.php) using the criteria of oral bioavailability (OB) ≥ 30% and drug-like properties (DL) ≥ 0.18. The targets of the screened components were obtained, and the corresponding genes of the human-related proteins were downloaded from the UniProt database (https://www.uniprot.org). After conversion using Perl scripts, the gene symbols of the active ingredients were obtained.

The string database (https://string-db.org/) was used to explore the protein-protein interactions (PPIs). Cytoscape software (version 3.9.1) was applied to construct the chemical-target network. Then, the selected 36 core targets were subjected to gene ontology (GO) enrichment analysis and Kyoto Encyclopedia of Genes and Genomes (KEGG) enrichment analysis using the DIVID database (david.ncifcrf.gov/). The microbiome online visualization tool (http://www.bioinformatics.com.cn/) was used for plotting.

### 2.7 Molecular simulation

The 2D structure of the active ingredient with proven SR activity was obtained through the PubChem database and imported into the Chem 3D software to obtain its 3D structure after energy minimization conversion. The crystal structures of the core target genes were downloaded separately from the PDB database (http://www.bioinformatics.com.cn/). Utilizing the Pymol software (Version 2.5.5), ligands and water molecules were removed through preprocessing to obtain a new 3D structure. Subsequently, Auto Dock Tools (Version 4.2) was used for hydrogenation, charge calculation, and other processing, and the results were exported in PDBQT format. Finally, Auto Dock Vina was used for molecular docking, and Discovery Studio Visualizer was used for result visualization. The Affinity (kcal/mol) value represents the binding ability of the two molecules. The Affinity < 0 indicates that molecules can freely bind. The first three with the lowest binding energy were used to analyze and observe the crucial results between the active ingredients and the target protein using Pymol software.

## 3 Results

### 3.1 UPLC-MS/MS component analysis

**Figure 2:**
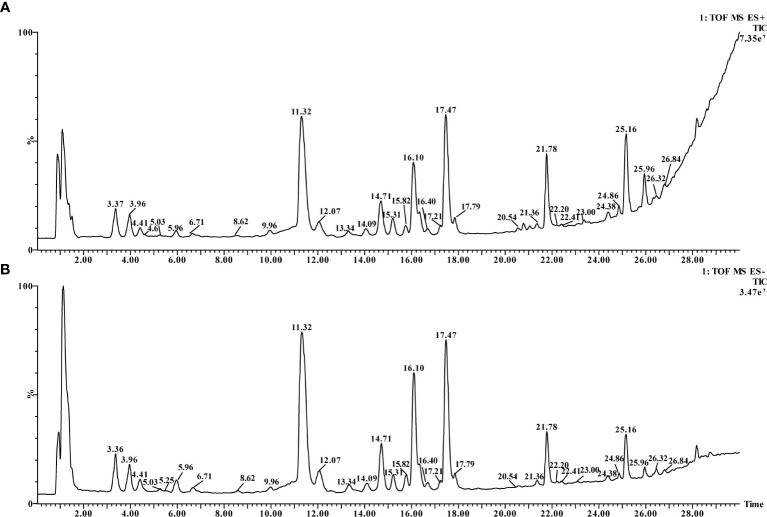
TIC of SR. **(A)** positive ion mode, **(B)** negative ion mode.

The negative ion mode complete scan total ion flow diagram (TIC) was shown in **Figure 2**. A Total of 35 components were identified from SR by positive and negative MS/MS ion fragments (**Table 3**), including flavonoids, terpenoids, and triterpenoid saponins (Islam et al., 2013; He et al., 2016; Hu et al., 2020).

**Table 3:** Tentative identification result of methanol extract of SR.

No.	t_R_/min	Ion mode	Formula	Calculate mass	Measured mass	δ/ppm	Fragment ions (m/z)	Identification results
1	3.37	[M+H]^+^ [M-H]^-^	C_26_H_28_O_13_	549.1608(+) 547.1452(-)	549.1611 547.1451	0.5 -0.2	(+) 495.1292, 411.1080, 393.0924, 375.0868, 363.0869 (-) 529.1329, 487.1234, 457.1132, 337.0706	Chrysin 6-C-arabinoside -8-C-glucoside
2	3.97	[M+H]^+^ [M-H]^-^	C_26_H_28_O_13_	549.1608(+)547.1452(-)	549.1611 547.1458	1.1 0.5	(+) 441.1122, 435.1080, 381.0975, 363.0868 (-) 116.9272, 281.0792, 337.0710, 427.1026	Chrysin 6-C-glucoside -8-C-arabinoside
3	4.47	[M+H]^+^ [M-H]^-^	C_26_H_28_O_13_	549.1608(+)547.1452(-)	549.1614 547.1451	1.1 -0.2	(+) 441.1122, 435.1081, 381.0974, 363.0873 (-) 457.1130, 427.1024, 337.0715, 299.0917	Chrysin 6-C-arabinoside -8-C-glucoside
4	4.62	[M+H]^+^	C_26_H_26_O_13_	547.1452(+)	547.1458	1.1	(+) 457.1133, 427.1024, 337.0712	7-O-ribofuranosyladriamycinone
5	5.03	[M+H]^+^ [M-H]^-^	C_23_H_24_O_13_	509.1295(+)507.1139(-)	509.1291 507.1140	-0.8 0.1	(+) 332.0539, 314.0429, 247.0765, 271.0596 (-) 345.0612, 330.0376, 241.8981, 197.0446	Viscidulin III-2’-O-glucoside
6	5.25	[M-H]^-^	C_21_H_20_O_12_	463.1877(-)	463.0878	0.1	(-) 287.0555, 164.9824, 153.0181	Carthamidin-7-O- glucoside isomer
7	5.98	[M+H]^+^ [M-H]^-^	C_15_H_12_O_7_	305.0661(+)303.0505(-)	305.0660 303.0506	-0.3 0.3	(+) 297.0759, 267.0648, 153.0182 (-) 125.0238, 177.0188, 217.0501, 295.0608	5-Hydroxy-2-hydroxy-flavone
8	6.7	[M+H]^+^ [M-H]^-^	C_21_H_18_O_12_	463.0877(+)461.0720(-)	463.0081 461.0728	0.9 1.7	(+) 340.0734, 331.0812, 287.0556 (-) 285.0400, 255.0293, 329.0660, 491.1177	Scutellarin
9	8.6	[M+H]^+^ [M-H]^-^	C_15_H_10_O_7_	303.0505(+) 301.0348(-)	303.0507 301.0351	0.2 0.3	(+) 229.0486, 153.0184 (-) 243.8983, 149.0231, 125.0236	3,5,7,2’,6’-Pentahydroxyflavone
10	9.96	[M+H]^+^ [M-H]^-^	C_21_H_20_O_10_	433.1135(+) 431.0978(-)	433.1135 431.0974	0.0 -0.9	(+) 271.0606 (-) 269.0452, 239.0343	Cosmosiin
11	11.32	[M+H]^+^ [M-H]^-^	C_21_H_18_O_11_	447.0927(+) 445.0771(-)	445.0787 447.0950	5.1 2.5	(+) 269.0452, 271.0119, 241.0502, 139.0031 (-) 267.0306, 269.0460, 169.0654, 251.0354, 223.0403	Baicalin
12	12.07	[M+H]^+^ [M-H]^-^	C_21_H_20_O_11_	449.1084(+) 447.0927(-)	449.1082 447.0919	-0.4 -1.8	(+) 273.0761, 169.0135, 271.0605 (-) 271.0605, 269.0450, 243.0654	Dihydrobaicalin
13	13.34	[M+H]^+^ [M-H]^-^	C_22_H_20_O_12_	477.1033(+) 475.0877(-)	477.1031 475.0872	-0.2 -0.5	(+) 301.0709, 286.0475 (-) 299.0551, 284.0318, 267.0288	7-O-glucuronide of 3-hydroxy-4’-methoxy-flavone
14	14.10	[M+H]^+^ [M-H]^-^	C_17_H_14_O_8_	347.0767(+) 345.0610(-)	347.0767 345.0614	0.0 0.4	(+) 332.0514, 314.0430, 289.0352, 169.0136 (-) 330.0376, 315.0141	Viscidulin III
15	14.71	[M+H]^+^ [M-H]^-^	C_21_H_18_O_11_	417.1186(+) 415.1029(-)	417.1180 415.1024	-1.4 -1.2	(+) 255.0659 (-) 252.0420	Apigenin-7-glucuronide
16	15.3	[M+H]^+^ [M-H]^-^	C_22_H_20_O_12_	477.1033(+) 475.0877(-)	477.1033 475.0872	0.0 -1.1	(+) 301.0712, 286.0477, 271.0604, 169.0132 (-) 299.0551, 284.0316, 282.1062, 269.0441	7-O-glucuronide of 3-hydroxy-4-methoxy-flavone
17	15.8	[M+H]^+^ [M-H]^-^	C_21_H_18_O_11_	447.0927(+) 445.0771(-)	447.0930 445.0767	0.7 -0.9	(+) 271.0607, 153.0188 (-) 269.0407, 151.0026	Norwogonin 8-O-β-D-glucuronide
18	16.10	[M+H]^+^ [M-H]^-^	C_22_H_20_O_11_	461.1084(+) 459.0927(-)	461.108 459.0939	3.0 2.6	(+) 285.0800, 270.0537 (-) 283.0612, 268.0376	Oroxylin A -7-O-glucuronide
19	16.40	[M+H]^+^ [M-H]^-^	C_22_H_20_O_12_	477.1033(+) 475.0877(-)	477.1039 475.0877	1.3 0.0	(+) 301.0720, 286.0495 (-) 299.0555, 284.0495	Trihydroxymethoxy-flavone-O-glucuronide
20	17.21	[M+H]^+^ [M-H]^-^	C_21_H_18_O_11_	447.0927(+) 445.0771(-)	447.0930 445.0771	0.7 0.0	(+) 271.0608 (-) 269.0449	Baicalein 6-O-β-D-glucuronide
21	17.47	[M+H]^+^ [M-H]^-^	C_22_H_20_O_11_	461.1084(+) 459.0927 (-)	461.1129 459.0942	3.3 9.8	(-) 283.0607, 268.0771, 175.0240 (+) 285.0807, 270.0547	Wogonoside
22	17.80	[M+H]^+^ [M-H]^-^	C_23_H_22_O_12_	491.1190(+) 489.1033(-)	491.1192 489.1036	0.4 0.3	(+) 315.0869, 285.0770 (-) 313.0712, 298.0479, 283.0600	6-Methoxywogonin-7-O-glucuronide
23	20.50	[M+H]^+^ [M-H]^-^	C_16_H_12_O_6_	299.0556(-) 301.0712(+)	301.0713 299.0552	0.3 -1.3	(+) 286.0481, 183.9999 (-) 284.0318, 146.9648	2’,5,7-Trihydroxy-8-methoxy flavone
24	21.36	[M+H]^+^ [M-H]^-^	C_15_H_10_O_5_	271.0606(+)269.0450(-)	271.0605 269.0449	-0.4 -0.4	(+) 271.0109, 241.0495, 139.0029 (-) 225.0546, 197.0593, 169.0644, 269.0446, 171.0442	Norwogonin
25	21.78	[M+H]^+^ [M-H]^-^	C_15_H_10_O_5_	271.0606(+) 269.0450(-)	271.0615 269.0459	3.3 3.3	(+) 169.0135, 123.0081, 139.0028, 253.0502 (-) 239.0135, 223.0395, 195.0445, 179.0493	Baicalein
26	22.2	[M+H]^+^ [M-H]^-^	C_16_H_12_O_6_	301.0712(+)299.0556(-)	301.0710 299.0553	-0.7 -1.0	(+) 286.0473, 271.0601, 167.0342 (-) 284.0317, 269.0443, 136.9872	Hispidulin
27	22.4	[M+H]^+^ [M-H]^-^	C_16_H_12_O_6_	301.0712(+)299.0556(-)	301.0707 299.0552	-1.7 -1.3	(+) 287.0550, 271.0599, 242.2303, 153.0185 (-) 285.0403, 269.0449, 267.0292, 151.0030	Trihydroxy-methoxy-flavone
28	23.0	[M+H]^+^ [M-H]^-^	C_16_H_12_O_6_	301.0712(+) 299.0556(-)	301.0713 299.0556	0.3 0.0	(+) 286.0474 283.0314	Trihydroxy-methoxy-isoflavone
29	24.4	[M+H]^+^ [M-H]^-^	C_18_H_16_O_7_	345.0974(+)343.0818(-)	345.0976 343.0822	0.6 1.2	(+) 286.2202, 284.0680, 242.2301, 197.0449 (-) 313.0346, 284.0317, 328.0582	Skullcapflavone
30	24.8	[M+H]^+^ [M-H]^-^	C_30_H_18_O_10_	539.0978(+) 537.0822(-)	539.0985 537.0834	1.3 2.2	(+) 285.0400, 315.0869 (-) 391.0457, 245.0087	8,8’’-Bibaicalein
31	25.16	[M+H]^+^ [M-H]^-^	C_19_H_18_O_8_	375.1080(+)373.0923(-)	375.1099 373.0930	5.1 1.9	(+) 345.0629, 227.0553 (-) 343.0454, 328.0219	Skullcapflavone II
32	25.96	[M+H]^+^ [M-H]^-^	C_16_H_12_O_5_	285.0763(+)283.0606(-)	285.0777 283.0608	4.9 0.7	(+) 270.0541, 168.0057 (-) 268.0373, 239.0340	Wogonin
33	26.3	[M+H]+ [M-H]-	C_17_H_14_O_6_	315.0869(+) 313.0712(-)	315.0869 313.0711	0.0 -0.3	(+) 286.2205 (-) 298.0477, 283.0242	5,8-Dihydroxy-6,7-dimethoxyflavone
34	26.4	[M+H]^+^ [M-H]^-^	C_15_H_10_O_4_	255.0657(+) 253.0501(-)	255.0659 253.0498	0.8 -1.2	(+) 153.0186, 242.2306 (-) 209.0598, 143.0492	Chrysin
35	26.8	[M-H]^-^	C_18_H_16_O_7_	343.0818(-)	343.0817	-0.3	(-) 328.0584, 313.0347, 298.0112, 270.0163	5,2’ -Dihydroxy-6,7,8- trimethoxyflavone

### 3.2 Optimization of ultrasonic extraction processing technology of SR

#### 3.2.1 Method validation

The calibration curves of baicalin, baicalein, wogonin, and wogonoside were constructed by plotting the peak areas (y) versus the concentrations (x). The following relationships had good linearity for the indicated concentration ranges. The results were shown in **Supplementary Table S3**. A recovery accuracy test determined the method’s accuracy, and the results showed that the recovery rates were 100%, 99.5%, 102%, and 101%, with RSD of 1.38%, 1.23%, 2.68%, and 1.18%, respectively.

#### 3.2.2 Single factor experiment

The results of single factor experiment showed that various factors significantly influenced the comprehensive score of indicator components. The total score exhibited a trend of initial increase and subsequent decrease as the methanol concentration rose, peaking at 80%. Therefore, three concentrations of methanol, namely 70%, 80%, and 90%, were chosen to optimize the response surface design of the extraction solvent. Similarly, three groups of 60 min, 75 min, and 90 min were selected for response surface optimization design. Three levels of soaking time, 0, 0.5, and 1 hour, were chosen for response surface optimization design. Three levels of solvent multiples, 80, 120, and 160, were selected for response surface optimization design.

#### 3.3.3 Experimental design of RSM

The response surface analysis scheme and experimental results were shown in **Supplementary Table S4**. The obtained regression equation was as follows: Y = 0.94 + 1.92×10^-3^ A + 4.511×10^-3^ B + 1.05×10^-3^ C + 0.011 D + 2.817×10^-3^ AB + 6.192×10^-3^ AC - 0.014 AD + 7.498×10^-3^ BC + 7.478×10^-3^ BD - 1.456×10^-3^ CD - 0.038 A^2^ - 5.651×10^-3^ B^2^ - 0.018 C^2^ - 0.031 D^2^. The variance analysis of each term in the regression equation was shown in **Table 4**. The results showed that the model design is significant (*P* < 0.01), and the lack of fit term had a *P* value of 0.5207 > 0.05, indicated a good fit between the response values and the predicted values. The quadratic effects of immersion time and methanol concentration have a *P* value of < 0.05, indicatied a significant influence on the comprehensive score of the target components.

**Table 4:** The analysis results of the variance of the regression equation terms (^*^ *P* < 0.05, ^**^ *P* < 0.01).

Source of variance	The average sum of squares	Degree of freedom	Mean square	*F-*Value	*P-*Value	significance
Model	0.017	14	1.21×10^-3^	3.95	0.0074	**
A	4.42×10^-5^	1	4.42×10^-5^	0.14	0.71	
B	2.44×10^-4^	1	2.44×10^-4^	0.8	0.3876	
C	1.32×10^-5^	1	1.32×10^-5^	0.043	0.8385	
D	1.46×10^-3^	1	1.46×10^-3^	4.75	0.0468	*
AB	3.17×10^-5^	1	3.17×10^-5^	0.1	0.7526	
AC	1.53×10^-4^	1	1.53×10^-4^	0.5	0.4913	
AD	8.06×10^-4^	1	8.06×10^-4^	2.62	0.1275	
BC	2.25×10^-4^	1	2.25×10^-4^	0.73	0.4065	
BD	2.24×10^-4^	1	2.24×10^-4^	0.73	0.4078	
CD	8.48×10^-6^	1	8.48×10^-6^	0.028	0.8704	
A2	9.43×10^-3^	1	9.43×10^-3^	30.71	< 0.0001	**
B2	2.07×10^-3^	1	2.07×10^-4^	0.67	0.4252	
C2	2.18×10^-3^	1	2.18×10^-3^	7.1	0.0185	*
D2	6.09×10^-3^	1	6.09×10^-3^	19.83	0.0005	**
Residual error	4.30×10^-3^	14	3.07×10^-4^			
Missing fit	3.12×10^-3^	10	3.12×10^-4^	1.06	0.5207	
Pure error	1.18×10^-3^	4	2.94×10^-4^			
Total value	0.021	28				

**Figure 3:**
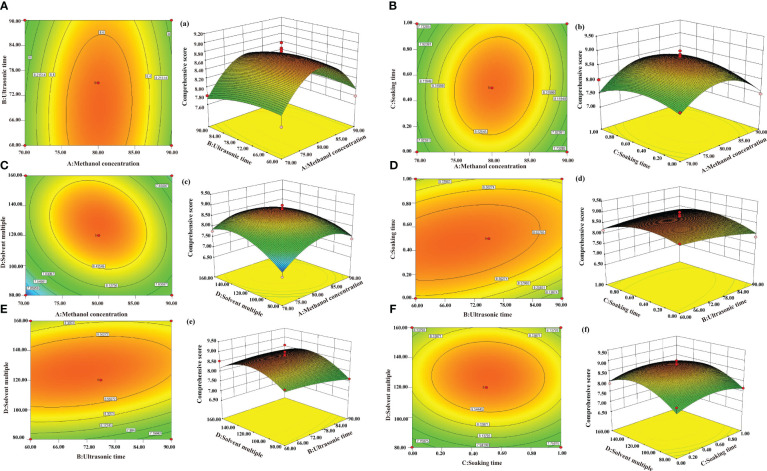
Contours and response surface 3D maps of the influence on the overall score. **(A–F)** Contour map; **(A–F)** Response surface 3D map.

The interaction contour and 3D surface diagram of AB, AC, AD, BC, BD, and CD were displayed in **Figure 3**. The optimal extraction process obtained was as follows: a methanol concentration of 79.85%, a solvent multiple of 120.57 times, a soaking time of 0.48 hour, and an ultrasonic time of 70.75 min. To facilitate the testing, the verification scheme was adjusted to confirm the following parameters: a methanol concentration of 80%, a solvent multiple of 120 times, a soaking time of 0.5 hours, and an ultrasonic time of 70 min. This adjustment proved that the process was stable and feasible.

### 3.4 Establishment of fingerprint

#### 3.4.1 Method validation

The retention time and peak area of all peaks with good separation were retrieved. The relative standard deviation (RSD) values for retention time did not exceed 0.90%, whereas that for peak areas was less than 2.54%, indicating good precision, repeatability, and stability.

#### 3.4.2 Establishment of UPLC fingerprint

**Figure 4:**
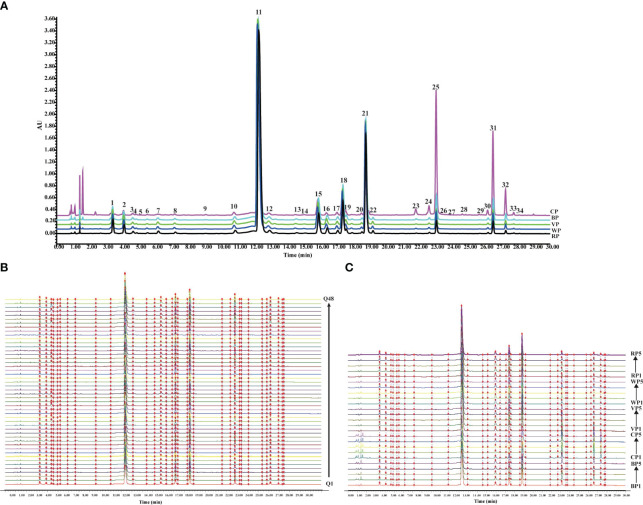
The chromatographic fingerprints of SR and different processed products. **(A)** SR raw product and UPLC chromatogram of four processed products. **(B)** The chromatographic fingerprints of 48 batches of SR. **(C)** The chromatographic fingerprints of five kinds of 25 batches of different processed products of SR.

The UPLC fingerprints of raw SR and four different processed products are shown in **Figure 4A**. There were 34 common peaks between different processed products and raw products, and there were significant differences in peak areas between different chromatographic peaks. For example, peaks 25, 31, and 32 in CP had significantly increased peak areas compared to raw and other prepared products.

#### 3.4.3 Analysis of similarity evaluation

The similarity results of 48 batches of SR were shown in **Supplementary Table S5**. And the similarity results of four kinds of prepared products and raw products were shown in **Supplementary Table S6**. And the fingerprints of 48 batches of SR and 25 batches of SR with different processing methods were shown in **Figures 4B, C**. The similarity of samples of different batches of SR was greater than 0.9, and the similarity of samples of each prepared product was also greater than 0.9. The result showed that the overall quality of SR from different sources and different products were relatively stable.

### 3.5 Multivariate chemometric analysis

#### 3.5.1 HCA and PCA

**Figure 5:**
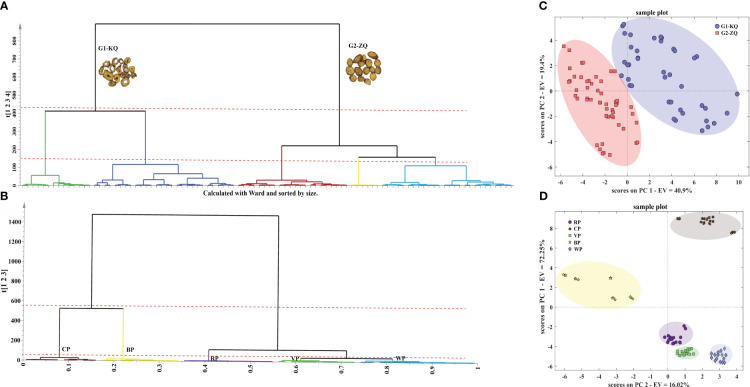
HCA and PCA plot of SR. **(A)** HCA plot of 48 batches SR; **(B)** HCA plot of different processed products of SR; **(C)** PCA plot of 48 batches SR; **(D)** PCA plot of different processed products of SR.

Ward’s method was then used for sequential clustering analysis on the samples. **Figure 5A** showed that when the distance scale was 400, the 48 batches of SR raw product samples can be clustered into two categories: KQ (G1) and ZQ (G2). When the scale was 200, the KQ could be further divided into two categories: originating from Neimenggu and Shanxi; the ZQ could be divided into three categories: arising from Neimenggu, Hebei, and Shanxi. Serial clustering analysis was conducted on the four self-made processed and raw products. The results were shown in **Figure 5B**. When the scale was 600, CP and BP samples cluster together, while RP, VP, and WP cluster together. When the scale is 50, both the raw products and the four types of processed products can cluster separately. The results showed that the growth age of SR was the main factor affecting the differences in its intrinsic chemical composition, and the model developed can firstly distinguish KQ and ZQ with different growth ages; whereas, the origin was the second factor contributing to the differences in its chemical composition, and the differences between different batches of KQ and ZQ can be further explored, and each of them can be distinguished according to the source of origin again. Moreover, the differences in their chemical compositions after the concoction treatment were obvious, and each could be distinguished from the other.

Preprocess the UPLC liquid phase raw data of the 48 batches of SR raw products using “autoscale” as the preprocessing method and obtain a data matrix (96 samples × 34 variables). Select eight principal components with eigenvalues greater than 1 to construct a PCA model, where PC1 and PC2 explain 40.9% and 19.4% of the variables, respectively. Plot the samples’ two-dimensional score map using PC1 and PC2 (**Figure 5C**). From the graph, it can be observed that the two types of samples were clustered separately. Similarly, we built PCA models for KQ and ZQ samples individually (**Supplementary Figures S1A, B**), and the classification results were consistent with HCA. Different chemical compositions were the primary factor that affects the quality of SR, as the SR forms had significant variations. The origin of the source may be the secondary factor that affects the active components of SR. A PCA model was established for raw materials and four types of processed products (**Figure 5D**). From the figure, it could be observed that each of the five sample categories forms a separate cluster. The samples of BP and CP were notably distant from the other samples, indicating that the degree of heating greatly influences the quality of SR. Additionally, the processed products with added auxiliary materials have chemical compositions closer to the raw materials.

#### 3.5.2 PLS-DA

**Figure 6:**
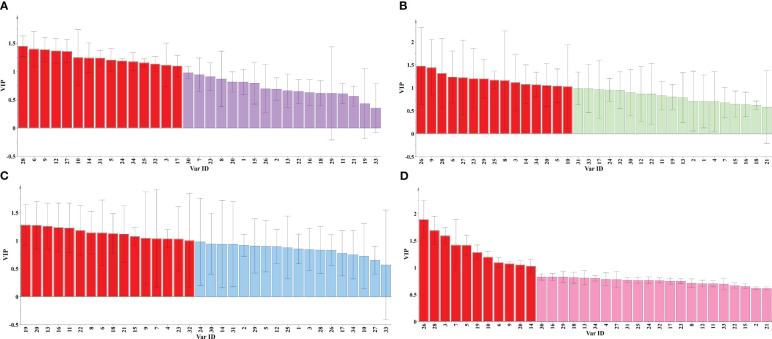
VIP plot of SR. **(A)** VIP plot of 48 batches SR; **(B)** VIP plot of KQ; **(C)** VIP plot of ZQ; **(D)** VIP plot of different processed products.

Supervised PLS-DA models had established SR further to verify the classification results of unsupervised pattern recognition. **Supplementary Figure S2** was the score plot of the PLS-DA model; the classification results were consistent with HCA and PCA. The VIP chart was drawn to clarify the importance of each variable for the classification (**Figure 6**). Among them, the variables 8 (scutellarin), 6 (carthamidin-7-O-glucoside isomer), 9 (3,5,7,2’,6’-pentahydroxy flavone), 12 (dihydro baicalin), 27 (trihydroxy-methoxy-flavone), 10 (cosmosiin), 14 (viscidulin III), 31 (skullcapflavone II), 5 (viscidulin III-2’-O-glucoside), 24 (norwogonin), 34 (chrysin), 25 (baicalein), 32 (wogonin), 3 (chrysin 6-C-arabinoside 8-C-glucoside), and 17 (norwogonin 8-O-β-D-glucuronide) could be used as essential chemical markers (VIP>1) to distinguish between KQ and ZQ. Additionally, the variables 26 (hispidulin), 9 (3,5,7,2’,6’-pentahydroxy flavone), 28 (trihydroxy-methoxy-isoflavone), 6 (carthamidin-7-O-glucoside isomer), 27 (trihydroxy-methoxy-flavone), 23 (2’,5,7-trihydroxy-8-methoxy flavone), 29 (skullcapflavone), 25 (baicalein), 8 (scutellarin), 3 (chrysin 6-C-arabinoside 8-C-glucoside), 14 (viscidulin III), 34 (chrysin), 20 (baicalein 6-O-β-D-glucuronide), 5 (viscidulin III-2’-O-glucoside), and 10 (cosmosiin) could be utilized as significant chemical markers to differentiate between the production areas of KQ (Neimenggu and Shanxi).

Furthermore, the variables 19 (trihydroxymethoxy-flavone-O-glucuronide), 20 (baicalein 6-O-β-D-glucuronid), 13 (7-O-glucuronide-3-hydroxy-4’-methoxy-flavone), 16 (7-O-glucuronide-3-hydroxy-4-methoxy-flavone), 11 (baicalin), 22 (6-methoxywogonin-7-O-glucuronide), 8 (scutellarin), 6 (carthamidin-7-O-glucoside isomer), 18 (oroxylin A-7-O-glucuronide), 21 (wogonoside), 15 (apigenin-7-glucuronide), 9 (3,5,7,2’,6’-pentahydroxy flavone), 7 (5-hydroxy-2-hydroxy-flavone), 4 (7-O-ribofuranosyladriamycinone), 23 (2’,5,7-trihydroxy-8-methoxy flavone), and 32 (wogonin) could be incorporated as essential chemical markers to distinguish between the production areas of ZQ (Neimenggu, Shanxi, and Hebei). Lastly, variables 26 (hispidulin), 28 (trihydroxy-methoxy-isoflavone), 3 (chrysin 6-C-arabinoside-8-C-glucoside), 7 (5-hydroxy-2-hydroxy-flavone), 5 (viscidulin III-2’-O-glucoside), 19 (trihydroxymethoxy-flavone-O-glucuronide), 10 (cosmosiin), 6 (carthamidin-7-O- glucoside isomer), 9 (3,5,7,2’,6’-pentahydroxy flavone), 20 (baicalein 6-O-β-D-glucuronid), and 14 (viscidulin III) played a crucial role as chemical markers to distinguish between four processed products and raw products.

#### 3.5.3 CP-ANN

**Figure 7:**
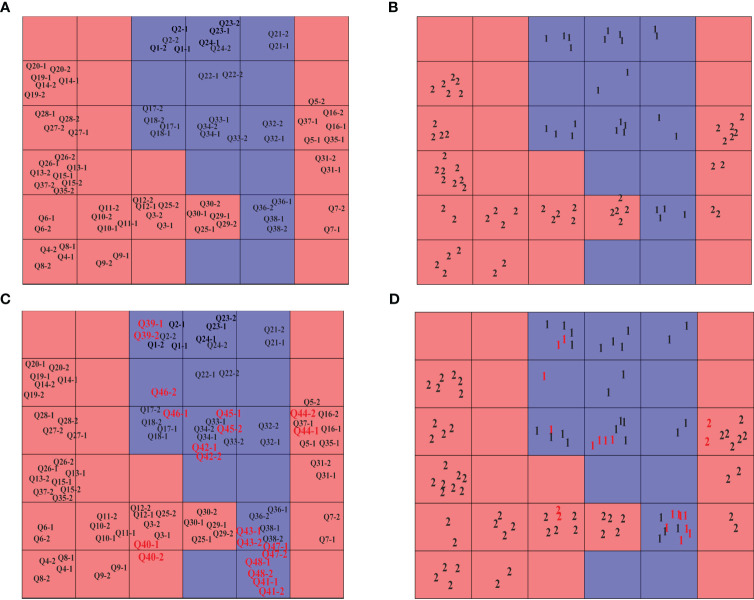
Kohonen map of 48 batches of SR and their classes. **(A)** modeling samples; **(B)** modeling classes; **(C)** forecasting samples; **(D)** forecasting classes.

According to the clustering results from HCA and PCA, a CP-ANN pattern recognition model was created using 48 batches of SR. The numbers 1 and 2 represent KQ and ZQ samples, respectively. The neural network structure of CP-ANN was optimized using a genetic algorithm, resulting in an optimal network structure consisting of 6×6 neurons and 200 iterations. The CP-ANN model was then established using these optimal parameters. The distribution of the used SR samples and their classes in the Kohonen map can be seen in **Figures 7A, B**, while the distribution of the predicted samples and their outcomes in the Kohonen map was shown in **Figures 7C, D**. It was clear that KQ and ZQ samples occupy separate neurons without any overlap, and the prediction error rate was 0, indicating a high level of accuracy in both model fitting and classification results. Furthermore, a CP-ANN model was established for raw SR samples and four types of processed SR samples. The distribution of the used samples and their classes in the Kohonen map were displayed in **Supplementary Figures S3A, B**, while the distribution of the predicted samples and their outcomes in the Kohonen map were shown in **Supplementary Figure S3C, D**. It could be seen that the four types of processed samples and raw samples occupy distinct neurons without any overlap.

**Figure 8:**
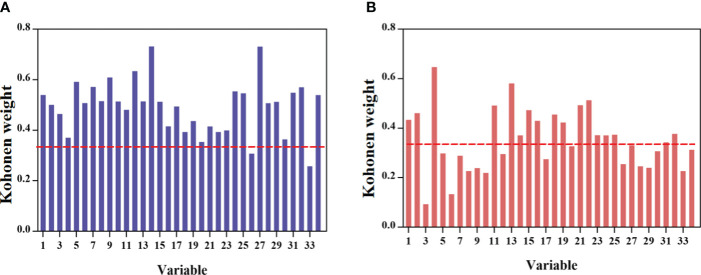
Kohonen weight of chemical components. **(A)** class 1-KQ; **(B)** class 2 -ZQ.

The weighting values of various variables were obtained through CP-ANN to assess the contribution rate of different variables in KQ and ZQ. Furthermore, a condition was imposed where the Kohonen weighting value exceeded 0.35 to identify the chemical markers of KQ and ZQ, as depicted in **Figures 8A, B**. It could be seen that 3 (chrysin 6-C- arabinoside-8-C-glucoside), 7 (5-hydroxy-2-hydroxy-flavone), 8 (scutellarin), 9 (3,5,7,2’,6’-pentahydroxy flavone), 10 (cosmosiin), 12 (dihydrobaicalin), 17 (norwogonin 8-O-β-D-glucuronide), 26 (hispidulin), 27 (trihydroxy-methoxy-flavone), 28 (trihydroxy-dimethoxy-isflavone), 29 (skullcapflavone), 30 (8,8’’-Bibaicalein), 31 (skullcapflavone II) are unique chemical markers of KQ. Variable 20 (baicalein 6-O-β-D-glucuronide) is a unique chemical marker of ZQ.

Obtained chemical markers for four types of prepared products and raw products based on the weight values using Kohonen’s method. As shown in **Supplementary Figure S4**, it could be seen that there are significant differences in the weight values of variables between different prepared products and raw products, especially with variable differences being most obvious in CP. Among them, variables 1 (chrysin 6-C-arabinoside-8-C-glucoside), 2 (chrysin 6-C-arabinoside-8-C-glucoside), 3 (chrysin 6-C-arabinoside-8-C-glucoside), 4 (7-O-ribofuranosyladriamycinone), 5 (viscidulin III-2’-O-glucoside), 6 (carthamidin-7-O-glucoside isomer), 8 (scutellarin), 10 (cosmosiin), 11 (baicalin), 12 (dihydrobaicalin), 13 (7-O-glucuronide of 3-hydroxy-4’-methoxy-flavone), 15 (apigenin-7-glucuronide), 16 (7-O-glucuronide of 3-hydroxy-4-methoxy-flavone), 18 (oroxylin A-7-O-glucuronide), and 21 (wogonoside) were unique markers of raw products compared to CP; variables 7 (5-hydroxy-2-hydroxy-flavone), 10 (cosmosiin), 14 (viscidulin III), 19 (trihydroxymethoxy-flavone-O-glucuronide), 20 (baicalein 6-O-β-D-glucuronide), 26 (hispidulin), 28 (trihydroxy-dimethoxy-flavone), and 29 (skullcapflavone) were unique markers of raw products compared to BP; variables 7 (5-hydroxy-2-hydroxy-flavone), 26 (hispidulin), 28 (trihydroxy-dimethoxy-flavone), and 29 (skullcapflavone) are unique markers of raw products compared to VP; variables 7 (5-hydroxy-2-hydroxy-flavone), 26 (hispidulin), 28 (trihydroxy-dimethoxy-flavone), and 29 (skullcapflavone), were unique markers of raw products compared to WP.

### 3.6 Target network analysis

**Figure 9:**
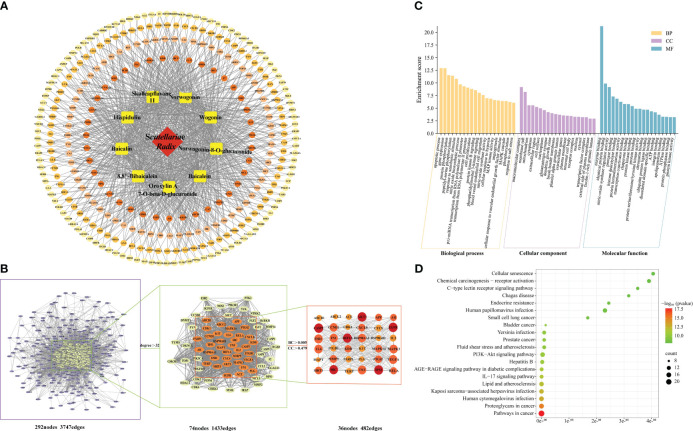
The target network of SR. **(A)** Network diagram of “SR-active component-target pathway”; **(B)** The PPI network; **(C)** GO Analysis of SR; **(D)** KEGG analysis of SR. The size and color were correlated to the degrees of targets in the network: the big size and deep color with purple means a high degree of this target.

According to the screening results of PLS-DA and CP-ANN, the chemical marker components affecting SR quality (processing, growth age, origin) were selected as candidate active ingredients for quality markers. Furthermore, TCMSP and other databases were searched, and using OB≥30% and DL ≥0.18 as criteria, baicalin, baicalein, wogonin, norwogonin, norwogonin-8-O-glucuronide, skullcap flavone II, hispidulin, 8, 8”- bibaicalein, and oroxylin A-7-O-beta-D-glucuronide were selected as the active ingredients of SR, and used as “Related Targets” to obtain the target of all active ingredients. The 292 target sites of SR were uploaded to the String database, and the PPI network was visualized by Cytoscape 3.9.1 software (**Figure 9A**). The network includes 292 nodes and 3747 edges, where nodes represent target gene proteins and edges illustrate interactions between proteins. The PPI Network (**Figure 9B**) was analyzed topologically through the Analyse Network plug-in, and the double median of degree was adopted as the screening condition; that is, the degree value was greater than 32, and a second network was obtained, which includes 74 nodes and 1433 edges. The core network was obtained by using the double median of Betweenness Centrality (BC) and Closeness Centrality (CC), that was, BC > 0.005 and CC > 0.479. The network consists of 36 nodes and 482 edges. The 36 core targets were sorted according to the degree value, among which AKT1, SRC, EGFR, CASP3, and TP53 were the top five, which could be used as key core targets (**Supplementary Table S7** **).**

GO enrichment analysis was displayed in bar graphs (**Figure 9C**), while KEGG enrichment analysis was shown in bubble plots (**Figure 9D**). The top 20 components with P-values are selected for plotting for GO enrichment analysis. The biological processes included apoptosis, gene expression, and protein phosphorylation. The cellular components included macromolecular complexes, mitochondria, and cytoplasm. The molecular functions included enzyme binding, nitric oxide synthase regulator activity, and protein binding. The KEGG pathway analysis results showed that 36 core targets are enriched in 129 pathways. The top 20 pathways were selected for display, including cancer pathways, human cytomegalovirus infection, kaposi sarcoma-associated herpesvirus infection, hepatitis B, and the IL-17 signaling pathway.

### 3.7 Molecular simulation

**Figure 10:**
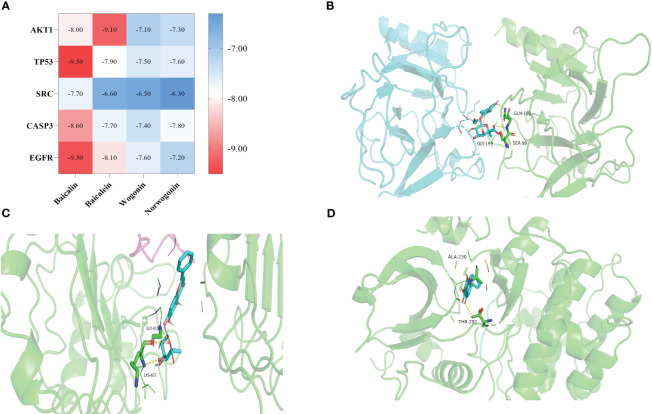
Molecular docking results. **(A)** Heat maps of molecular docking results; **(B)** Molecular docking pattern of baicalin and TP53; **(C)** Molecular docking pattern of baicalin and EGFR; **(D)** Molecular docking pattern of baicalein and AKT1.

The five essential target proteins screened out were scored by docking with the active ingredients (baicalin, baicalein, wogonin, norwogonin) in SR. The docking results were shown in **Supplementary Table S8**, and the thermal mapping software was used for visual analysis of the molecular docking results, as shown in **Figure 10**. The results showed that the affinity between baicalin and each target was better, and the binding energy was lowest. Compared with the other four targets, the binding energy between EGFR and the active ingredient was lowest and the binding power was strongest.

## 4 Discussion

The research on evaluating the quality of TCM is crucial for measuring, controlling, and ensuring its quality. In the modernization and internationalization of TCM, the quality of Chinese medicinal materials is a critical issue that requires attention. However, the current chemical markers used for quality evaluation of TCM predominantly focus on a singular assessment of chemical components, overlooking the inherent chemical complexities and the multifaceted pharmacological activity mechanisms. In some instances, certain currently selected markers are unsuitable for the comprehensive evaluation of TCM preparations that comprise numerous constituents (Wang et al., 2020). Furthermore, the quality of TCM is influenced by various factors such as its species, origin, growth duration, processing methods, storage conditions, and more. Therefore, it is urgent to establish a comprehensive quality evaluation method that aligns with the current research status. Q-markers, whose concept was proposed by academician Liu Changxiao, are of great significance for standardizing the quality control system of TCM (Yang et al., 2024).

SR, a commonly used medicinal herb in TCM, has a close relationship between its quality control and clinical efficacy. The origin of SR is diverse, with distributions covering most regions in northern China. Additionally, SR is categorized into KQ and ZQ based on their growth duration. However, there are currently limited studies on the quality evaluation and effectiveness, resulting in a lack of systematic evaluations. Moreover, there are numerous processing methods for SR, and different processing techniques can lead to changes in its active ingredients, thereby altering its medicinal properties. Clarifying the influence of processing on the medicinal properties of TCM is a prerequisite for ensuring clinical efficacy.

Maximizing the extraction of medicinal ingredients is of great significance for ensuring clinical efficacy and improving drug utilization efficiency. RSM is a widely used method for optimizing experimental conditions in recent years (De Leon et al., 2010; Kumari et al., 2021). The single-factor experiment combined with Box-Behnken Design was adopted to determine the optimal extraction process. This provides technical support for the extraction and further development and utilization of effective substances in SR. UPLC-Q-TOF-MS is one of the most effective methods for the analysis and identification of multi-component in complex matrices in recent years (Yin et al., 2006; Wang et al., 2013; Gao et al., 2017). In this study, UPLC-Q-TOF-MS was used for qualitative analysis of SR samples, identifying 35 compounds in total. The fingerprint chromatogram of TCM is one of the most effective ways recognized to control the quality. A UPLC fingerprint chromatogram of SR samples from different batches and prepared by different methods has been established, which has identified 34 common peaks. The method for the fingerprint chromatogram has been tested, and the results indicate that the analytical method is stable and reliable, meeting the requirements for fingerprint chromatogram measurement.

In order to further explore the differences among its internal components, a variety of artificial intelligence discrimination models have been constructed to objectively analyze the data obtained from the analysis, quantify the entire chromatogram information, and make it recognizable and processable by computers (Wang et al., 2024). The results show that 48 batches of SR can be grouped into two major categories: KQ and ZQ, which have great differences in chemical compositions and are the primary factor affecting the quality of SR. Further analysis shows that the samples of KQ and ZQ can be further distinguished based on their origins, indicating that the origin is a secondary factor affecting their chemical compositions. In addition, there are significant differences in chemical compositions among different processed products of SR, which can be clustered separately, especially the CP. This indicates that the degree of heating has a greater impact on the chemical compositions of SR compared to the addition of auxiliary materials. Therefore, attention should be paid to controlling the time and temperature during the processing. Finally, by considering the growth period, origin, and processing factors, we screened out chemical markers through the established CP-ANN and PLS-DA models, which can be used to comprehensively evaluate the quality of SR.

Network pharmacology utilizes searches through databases such as proteomics, genomics, and bioinformatics to conduct systematic analysis of TCM at both molecular and holistic levels. Based on network pharmacology, this study further screened out 8 key components and 292 targets by considering OB and DL. By constructing a protein-protein interaction network, 5 key targets of the 8 crucial compounds were identified: AKT1, SRC, EGFR, CASP3, and TP53. The AKT family kinases are indispensable and important components in the downstream activation of growth factor signaling pathways mediated by membrane-bound phosphoinositide-3 kinase (Marquard and Jücker, 2020). AKT1 plays a promotional role in epithelial-to-mesenchymal transition, invasion, disruption of tumor endothelial barrier, and cancer metastasis in cancer cells (Alwhaibi et al., 2019). CASP3 is the primary executor of apoptosis, converging both the intrinsic and extrinsic apoptotic pathways. Studies have shown that upregulating CASP3 can inhibit the proliferation, migration, and invasion of cancer cells, while promoting apoptosis (Lin et al., 2011; Zhao et al., 2019a). EGFR can directly participate in regulating the transcription of target genes as a transcription factor, and the mesenchymal-epithelial transition factor (MET) is closely related to the malignant phenotype of cancer cells (Bhushan et al., 2019). EGFR is also a TGF-α receptor, so it is speculated that the active ingredients in SR can block the binding of proinflammatory cytokines to EGFR by interacting with EGFR, thus exerting anti-inflammatory effects. And the overexpression of SRC promotes the progression of hepatocellular carcinoma, and inhibiting the expression of SRC significantly suppresses the proliferation of liver cancer cells (Jin et al., 2022). TP53 can regulate cell apoptosis and cell cycle arrest, and it works with the p300 gene to inhibit the activation of nuclear factor κB, Toll-like receptor 4, and ubiquitin ligase TRAF6, negatively regulating the secretion of inflammatory factors (Wang et al., 2015). GO enrichment analysis revealed that the gene functions of the active ingredients in SR mainly involve gene expression, protein phosphorylation, itric oxide synthase regulator activity, and protein binding. The key active ingredients of SR can exert anti-tumor and anti-inflammatory effects by mediating signaling pathways such as cancer pathways, hepatitis B, and the IL-17 signaling pathway through key target proteins including AKT1, EGFR, CASP3, SRC, and TP53.

The results of molecular docking showed that the key targets AKT1, EGFR, CASP3, SRC, and TP53 bind stably with the main active ingredients in SR, including baicalein, baicalin, wogonin, and wogonoside. Among the listed key targets, EGFR has excellent binding activity, which can be the focus of subsequent research on the anti-tumor and anti-inflammatory activities of SR. Therefore, the selected marker components can reflect the quality of SR and are associated with its functional activity, which can be used as Q-markers.

## 5 Conclusion

This investigation utilized UPLC-Q-TOF-MS combined fingerprint, artificial neural network, screening of characteristic components, network pharmacology, and molecular simulation to evaluate the quality of SR comprehensively. The results demonstrated that baicalin, baicalein, wogonin, norwogonin, norwogonin-8-O-glucuronide, skullcapflavone II, hispidulin, 8,8”-bibaicalein, and oroxylin A-7-O-beta-D-glucuronide can serve as Q-markers, reflecting the comprehensive effects of growth years, origin, and processing on SR, as well as their correlation with activity and efficacy. We have established a comprehensive evaluation model for the quality of SR that integrates TCM chemical composition, pharmacological activity, and efficacy. This model effectively enhances the quality standards of SR, ensuring the quality of Chinese medicinal materials and promoting the modernization development of TCM.

## Data availability statement

The data that support the findings of this study are openly available in [Figshare. Dataset] at https://doi.org/10.6084/m9.figshare.26122531.v1, reference number 26122531.v1.

## Author contributions

ML: Formal analysis, Writing – original draft. XZ: Writing – review & editing, Methodology. JW: Writing – review & editing, Methodology. LS: Writing – review & editing, Software. RH: Writing – review & editing, Software. HZ: Writing – review & editing, Methodology. YL: Writing – review & editing, Resources. XR: Writing – original draft, Project administration.
