# Supplementary material for: A multidimensional strategy for uncovering comprehensive quality markers of Scutellariae Radix based on UPLC-Q-TOF-MS analysis, artificial neural network, network pharmacology analysis, and molecular simulation
Source: Front Plant Sci. 2024 Jul 3;15:1423678. doi: 10.3389/fpls.2024.1423678 (PMC11251886; doi:10.3389/fpls.2024.1423678)
Supplement: Supplementary file 1 [file DataSheet_1.docx]

**Figure S1.** PCA plot of KQ (a) and ZQ (b).

**
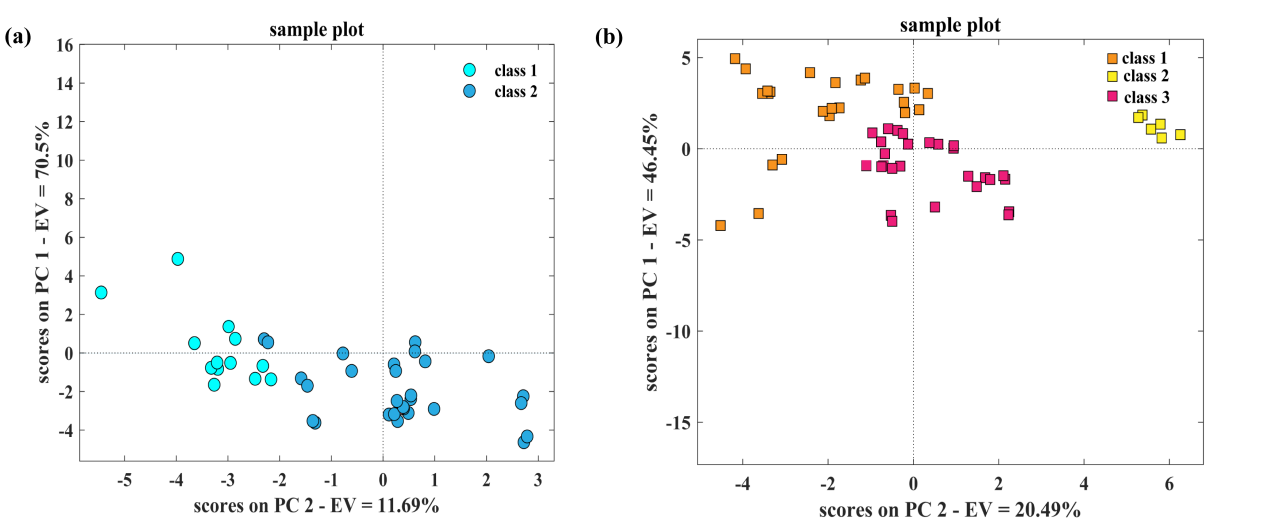
**

**Figure S2.** PLS-DA plot of SR. (a) PLS-DA plot of 48 batches SR; (b) PLS-DA plot of KQ; (c)PLS-DA plot of ZQ; (d) PLS-DA plot of different processed products.

**
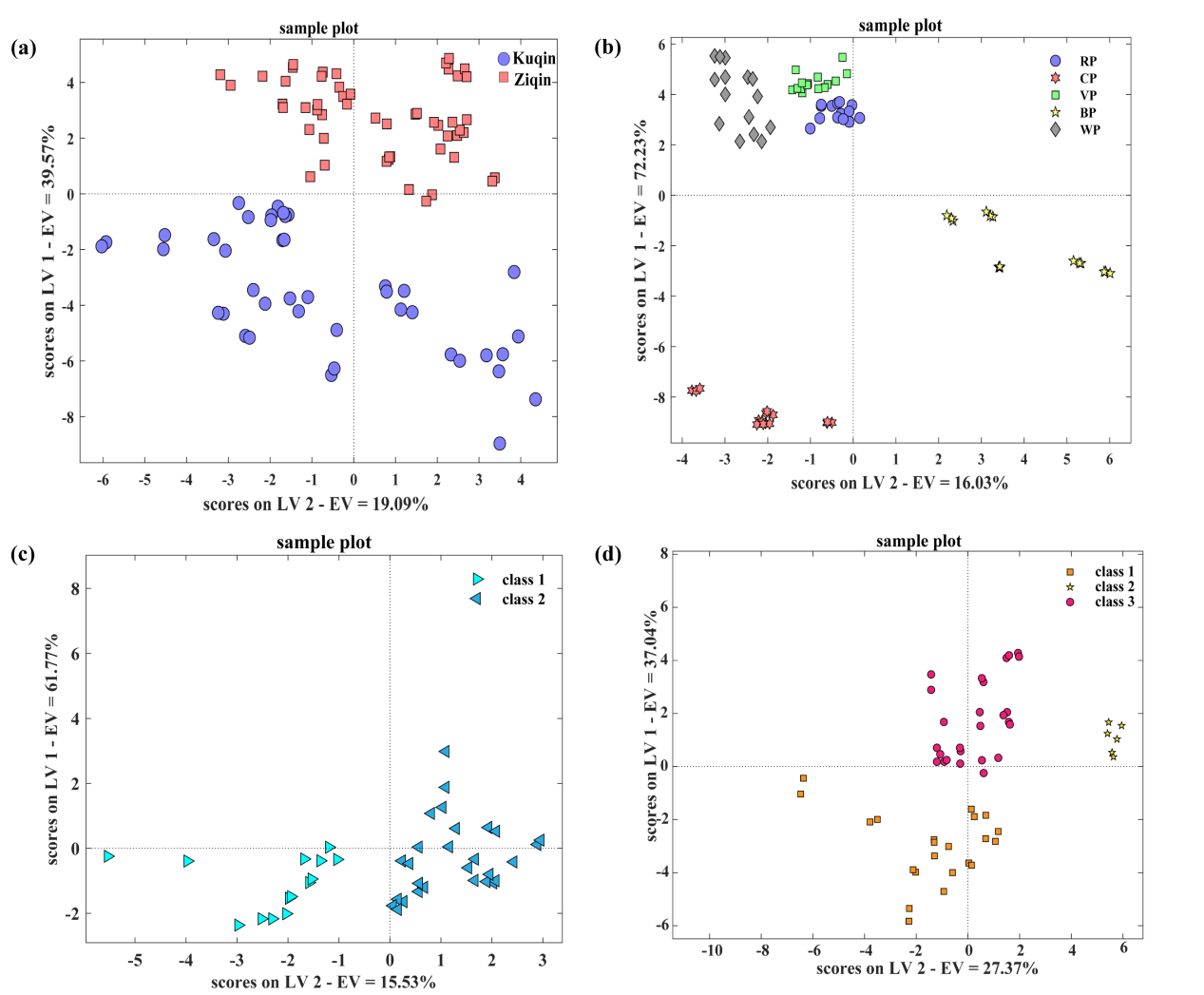
**

**Figure S3.** Konohen map of different processed products of SR and their classes. (a) modeling samples, (b) modeling classes, (c) forecasting samples, (d) forecasting classes.

**
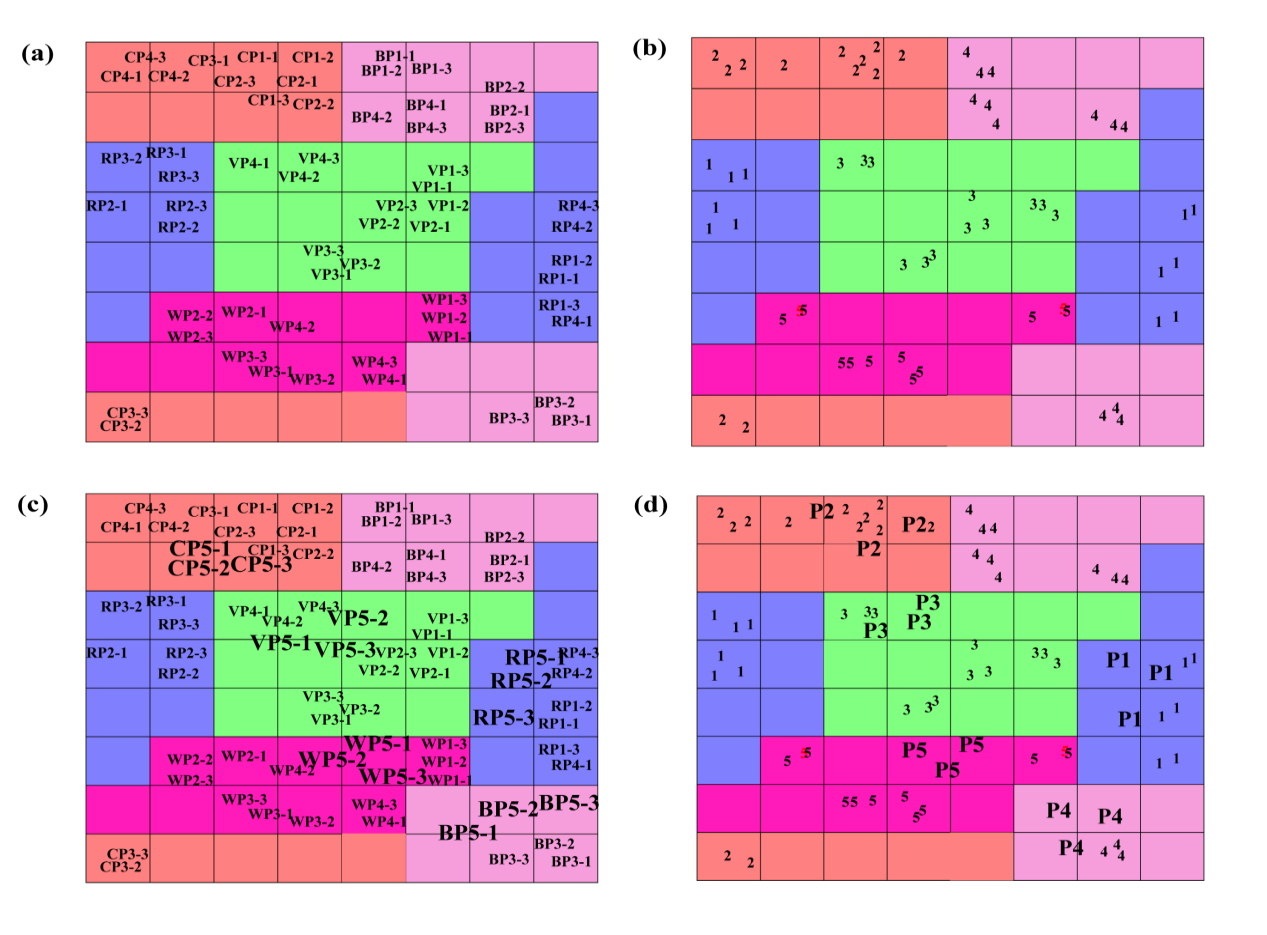
**

**Figure S4.** Konohen weight of chemical components for processed products of SR. (a) class 1 - RP; (b) class 2 - CP; (c) class 3 - VP; (d) class 4 - BP; (e) class5 - WP.

**
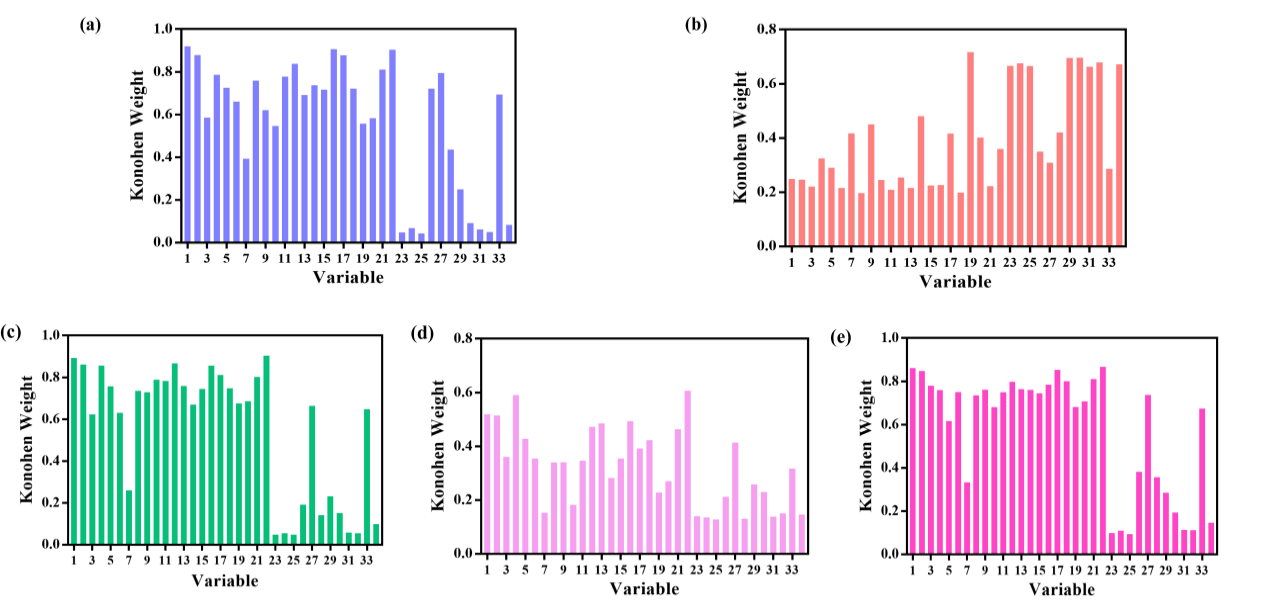
**

**Table S1**. Single factor experimental design.

| Factor | Methanol concentration (%) | Solvent multiple | Soaking time (h) | Ultrasonic time (min) |
| --- | --- | --- | --- | --- |
| Methanol concentration (%) | X_1_ | 80 | 0h | 30min |
| Solvent multiple | 80% | X_2_ | 0h | 30min |
| Soaking time (h) | 80% | 80 | X_3_ | 30min |
| Ultrasonic time (min) | 80% | 80 | 0h | X_4_ |

**Table S2.** Response surface analysis factors and horizontal design.

| Factor | Horizontal design | | |
| --- | --- | --- | --- |
|  | -1 | 0 | 1 |
| A-Methanol concentration (%) | 70 | 80 | 90 |
| B -Ultrasonic time (min) | 60 | 75 | 90 |
| C- Soaking time (h) | 0 | 0.5 | 1 |
| D- Solvent multiple) | 80 | 120 | 160 |

**Table S3.** Linear regression equation, correlation coefficient and linear range of baicalin, baicalein, wogonin, and wogonoside**.**

| **Compounds** | **equation of regression** | **r** | **Linear ranges (μg/mL)** |
| --- | --- | --- | --- |
| **Baicalin** | Y = 12034X +294020 | 0.9998 | 10.00-2000 |
| **Baicalein** | Y = 46434X + 7487.4 | 0.9997 | 1.56-100 |
| **Wogonin** | Y =50353X + 176344 | 0.9996 | 1.56-500 |
| **Wogonoside** | Y = 31801X - 384414 | 0.9993 | 3.13-500 |

**Table S4**. Response surface analysis scheme and test results.

| **Test** | **A-Methanol concentration (%)** | **B-****Ultrasonic time (min)** | C-Soaking time (h) | **D-****Solvent multiple** | **Comprehensive score** |
| --- | --- | --- | --- | --- | --- |
| 1 | 80 | 90 | 0.5 | 80 | 7.6307 |
| 2 | 90 | 75 | 0.5 | 80 | 7.4022 |
| 3 | 80 | 75 | 0 | 160 | 7.8992 |
| 4 | 80 | 75 | 0 | 80 | 7.8255 |
| 5 | 80 | 75 | 0.5 | 120 | 9.0007 |
| 6 | 70 | 75 | 0.5 | 80 | 6.7935 |
| 7 | 80 | 75 | 0.5 | 120 | 8.8099 |
| 8 | 80 | 90 | 0 | 120 | 7.8416 |
| 9 | 80 | 60 | 0.5 | 80 | 8.1489 |
| 10 | 80 | 75 | 1 | 160 | 7.6382 |
| 11 | 80 | 75 | 0.5 | 120 | 8.8847 |
| 12 | 80 | 60 | 0.5 | 160 | 8.5370 |
| 13 | 70 | 75 | 0 | 120 | 7.7921 |
| 14 | 80 | 75 | 1 | 80 | 7.6691 |
| 15 | 80 | 90 | 0.5 | 160 | 8.5641 |
| 16 | 80 | 60 | 0 | 120 | 8.5411 |
| 17 | 70 | 60 | 0.5 | 120 | 7.7509 |
| 18 | 90 | 75 | 0 | 120 | 7.4782 |
| 19 | 80 | 60 | 1 | 120 | 8.1558 |
| 20 | 90 | 60 | 0.5 | 120 | 7.7953 |
| 21 | 80 | 75 | 0.5 | 120 | 8.1513 |
| 22 | 80 | 90 | 1 | 120 | 8.0232 |
| 23 | 70 | 90 | 0.5 | 120 | 7.8045 |
| 24 | 90 | 75 | 1 | 120 | 8.1136 |
| 25 | 70 | 75 | 0.5 | 160 | 7.7685 |
| 26 | 90 | 90 | 0.5 | 120 | 8.0555 |
| 27 | 70 | 75 | 1 | 120 | 7.9855 |
| 28 | 80 | 75 | 0.5 | 120 | 8.8558 |
| 29 | 90 | 75 | 0.5 | 160 | 7.4272 |

**Table S5.** Similarity evaluation of 48 batches of SR.

| **Batches** | **Similarity** | **Batches** | **Similarity** | **Batches** | **Similarity** | **Batches** | **Similarity** |
| --- | --- | --- | --- | --- | --- | --- | --- |
| Q1 | 0.999 | Q13 | 0.999 | Q25 | 0.999 | Q37 | 1.000 |
| Q2 | 0.999 | Q14 | 0.998 | Q26 | 1.000 | Q38 | 0.999 |
| Q3 | 1.000 | Q15 | 0.999 | Q27 | 0.999 | Q39 | 0.999 |
| Q4 | 0.999 | Q16 | 0.999 | Q28 | 0.999 | Q40 | 0.999 |
| Q5 | 1.000 | Q17 | 0.997 | Q29 | 0.999 | Q41 | 1.000 |
| Q6 | 1.000 | Q18 | 0.998 | Q30 | 0.999 | Q42 | 0.994 |
| Q7 | 0.999 | Q19 | 0.997 | Q31 | 0.999 | Q43 | 0.999 |
| Q8 | 0.999 | Q20 | 0.997 | Q32 | 0.999 | Q44 | 1.000 |
| Q9 | 1.000 | Q21 | 0.998 | Q33 | 0.999 | Q45 | 0.997 |
| Q10 | 1.000 | Q22 | 0.999 | Q34 | 0.999 | Q46 | 0.995 |
| Q11 | 1.000 | Q23 | 0.999 | Q35 | 1.000 | Q47 | 0.999 |
| Q12 | 1.000 | Q24 | 0.999 | Q36 | 0.999 | Q48 | 0.999 |

**Table S6.** Similarity evaluation of different processed products of SR.

| **Batches** | **Similarity** | **Batches** | **Similarity** | **Batches** | **Similarity** | **Batches** | **Similarity** | **Batches** | **Similarity** |
| --- | --- | --- | --- | --- | --- | --- | --- | --- | --- |
| RP1 | 0.996 | BP1 | 0.999 | CP1 | 0.933 | VP1 | 0.997 | WP1 | 0.997 |
| RP2 | 0.997 | BP2 | 1.000 | CP2 | 0.925 | VP2 | 0.997 | WP2 | 0.997 |
| RP3 | 0.996 | BP3 | 0.999 | CP3 | 0.929 | VP3 | 0.997 | WP3 | 0.997 |
| RP4 | 0.996 | BP4 | 0.998 | CP4 | 0.927 | VP4 | 0.997 | WP4 | 0.998 |
| RP5 | 0.997 | BP5 | 1.000 | CP5 | 0.919 | VP5 | 0.997 | WP5 | 0.998 |

**Table S7. Core target sequencing for network pharmacological screening.**

| **Targets** | **Degree** | **Betweenness** | **Closeness** |
| --- | --- | --- | --- |
| **AKT1** | **147** | **0.088638917** | **0.652466368** |
| **TP53** | **142** | **0.080784938** | **0.638157895** |
| **SRC** | **135** | **0.056071616** | **0.628509719** |
| **CASP3** | **126** | **0.045953414** | **0.615221987** |
| **EGFR** | **114** | **0.045722012** | **0.601239669** |
| **CCND1** | **113** | **0.043349191** | **0.596311475** |
| **KDR** | **113** | **0.041703801** | **0.595092025** |
| **KIT** | **110** | **0.038224533** | **0.592668024** |
| **MAPK3** | **107** | **0.030331541** | **0.584337349** |
| **HSP90AA1** | **104** | **0.029263528** | **0.577380952** |
| **FN1** | **102** | **0.025650197** | **0.577380952** |
| **PTGS2** | **101** | **0.022413599** | **0.572834646** |
| **HSP90AB1** | **87** | **0.019541449** | **0.554285714** |
| **ABCG2** | **83** | **0.018341842** | **0.550094518** |
| **RELA** | **82** | **0.017675125** | **0.549056604** |
| **TNF** | **81** | **0.016314012** | **0.541899441** |
| **ESR1** | **75** | **0.015951617** | **0.541899441** |
| **HIF1A** | **69** | **0.015827286** | **0.536900369** |
| **ABCB1** | **65** | **0.01544198** | **0.523381295** |
| **VEGFA** | **63** | **0.013827846** | **0.523381295** |
| **AR** | **62** | **0.013798542** | **0.521505376** |
| **ITGB1** | **61** | **0.013690178** | **0.519642857** |
| **ACE** | **61** | **0.013477075** | **0.519642857** |
| **SIRT1** | **60** | **0.012453656** | **0.517793594** |
| **APP** | **59** | **0.012344446** | **0.515957447** |
| **CXCL8** | **59** | **0.011807928** | **0.514134276** |
| **MMP9** | **59** | **0.011143183** | **0.514134276** |
| **PLG** | **58** | **0.011138882** | **0.513227513** |
| **MAPT** | **58** | **0.010985797** | **0.513227513** |
| **MAPK14** | **56** | **0.010643754** | **0.512323944** |
| **IL2** | **55** | **0.010383459** | **0.512323944** |
| **CYCS** | **55** | **0.009837555** | **0.51142355** |
| **TERT** | **55** | **0.008756029** | **0.509632224** |
| **PARP1** | **53** | **0.008688002** | **0.505208333** |
| **CDK1** | **53** | **0.008504573** | **0.504332756** |
| **IL6** | **52** | **0.008301808** | **0.501724138** |

**Table S8.** Results of docking between the active ingredient and core protein of SR.

| **Composition** | **target** | **PDB ID** | **Affinity (kcal/mol)** |
| --- | --- | --- | --- |
| Baicalin | AKT1 | 3CQU | -8 |
|  | TP53 | 6GGC | -9.5 |
|  | SRC | 1O43 | -7.7 |
|  | CASP3 | 4PS0 | -8.6 |
|  | EGFR | 1mox | -9.3 |
| Baicalein | AKT1 | 3CQU | -9.1 |
|  | TP53 | 6GGC | -7.9 |
|  | SRC | 1O43 | -6.6 |
|  | CASP3 | 4PS0 | -7.7 |
|  | EGFR | 1mox | -8.1 |
| Wogonin | AKT1 | 3CQU | -7.1 |
|  | TP53 | 6GGC | -7.5 |
|  | SRC | 1O43 | -6.5 |
|  | CASP3 | 4PS0 | -7.4 |
|  | EGFR | 1mox | -7.6 |
| Norwogonin | AKT1 | 3CQU | -7.3 |
|  | TP53 | 6GGC | -7.6 |
|  | SRC | 1O43 | -6.3 |
|  | CASP3 | 4PS0 | -7.8 |
|  | EGFR | 1mox | -7.2 |
